# An experimental study on the curing of desert sand using bio-cement

**DOI:** 10.1186/s40643-024-00788-y

**Published:** 2024-07-20

**Authors:** Xiao Fu, Wan-jun Ye

**Affiliations:** School of Architecture and Civil Engineering, Xi‘an University of Science and Technology, Xi‘an, 710054 China

**Keywords:** Bio-cement, Tap water, Desert sand, Fly ash, Mechanical properties, Calcium carbonate precipitate

## Abstract

In order to promote the development and utilization of desert sand, this study is based on researching the most suitable ratio of bio-cement, analyzing the shear strength and permeability of improved desert sand by combining bio-cement and fly ash, and clarifying the applicability of tap water in bio-cement. The relationship between the two and the microstructural properties was investigated using the results of the straight shear test and the permeability test. The results showed that the urease solution prepared with tap water had a more pronounced temperature resistance. The urea concentration and the corresponding pH environment had a direct effect on the urease activity. The calcium carbonate yield was positively correlated with the calcium concentration, and the urea concentration was higher in the ranges of 1.0–1.5 mol/L. As the enzyme-to-gel ratio decreased, the calcium carbonate precipitate produced per unit volume of urease solution gradually converged to a certain value. The shear strength (increased by 37.9%) and permeability (decreased by about 8.9–68.5%) of the modified desert sand peaked with the increase in fly ash content. The microscopic test results indicated that the fly ash could provide nucleation sites for the bio-cement, effectively improving the mechanical properties of the desert sand. The crystal types of calcium carbonate in the modified desert sand were calcite and aragonite, which were the most stable crystal types. This study provides innovative ideas for interdisciplinary research in the fields of bioengineering, ecology and civil engineering.

**Supplementary Information:**

The online version contains supplementary material available at 10.1186/s40643-024-00788-y.

## Introduction

Deserts occupy a vast area of distribution in the world, and prolonged drought and exposed desert terrain seriously endanger the major challenges of human survival and sustainable development, accelerating the deterioration of the ecological environment and leading to the destruction of vegetation cover, soil erosion and the reduction of arable land resources. At the same time, the frequency and intensity of sandstorms and dust storms have increased, limiting to a certain extent the rapid socio-economic development. According to statistics, the direct economic losses caused by the sand and dust storm problem are over 100 million per year. Natural disasters such as dust storms and sand dune flows have brought ecological green development to a standstill, reduced the area of usable arable land in the countryside, and triggered multifaceted problems such as traffic congestion in cities, erosion of highway beds and burial of road cover by dust, all of which are serious hazards to human production, life and health (Sun et al. 2021a; Nieć et al. 2014). Effective management and rational utilization of desert sand is necessary for sustainable development. At present, many engineers and researchers have conducted in-depth studies on the physical, chemical and mechanical properties of desert sand and have achieved remarkable results, proving the usability of desert sand. The rational use of desert sand has the following advantages: (1) Promoting the development of silicate materials to low-cost and green manufacturing; (2) Previous investigations have revealed that desert sand products (Li et al. 2020; Liu et al. 2020) have comparable or even superior performance to traditional products; (3) The rational use of desert sand is capable of saving mineral resources (Wang et al. 2019; Benmerioul et al. 2017); (4) Improving the living standards of the inhabitants of desert areas. It can be seen that the utilisation of desert sand is necessary and has a strong potential, and desert sand products have become valuable industrial products.

With the significant increase in global energy demand and greenhouse gas emissions, the development of carbon-neutral sustainable energy sources and methods is encouraged. In this context, how can the mechanical properties of desert sands be improved and rationally utilised using green and ecological methods? Traditional ideas of chemical remediation are limited and also have pollution risks for the surrounding environment, which do not meet the requirements of environmental friendliness (Tao et al. 2021). Currently, a new technology for inducing carbonate precipitation has provided a new idea for soil improvement, which causes calcium carbonate precipitation to fill the pores between soil particles and bond soil particles to form aggregates, thereby improving the soil strength (Meng et al. 2021; Ahenkorah et al. 2021b, c; Min et al. 2020). Bio-cement has been utilized in many practical situations, including improving the strength of sandy soils (Al Imran et al. 2021) and clay soils (Moghal et al. 2020a), reducing soil conductivity (Almajed et al. 2020), stabilizing wind erosion (Sun et al. 2021b), resisting rain erosion (Moghal et al. 2020b), and removing heavy metal ions (Almajed et al. 2019). However, the lack of nucleation sites is a recognized shortcoming of Bio-cement (Enzyme-induced calcium carbonate precipitation, EICP) technology, which inevitably affects the soil improvement effect (Martin et al. 2021). In order to solve the problem of missing nucleation sites, researchers added additives to the improvement process to improve the overall improvement level of EICP (such as lignin, skimmed milk powder, and biopolymers (Sun et al. 2023; Zhang et al. 2022; Arab et al. 2021)). As one of the main by-products of coal combustion, fly ash is an industrial waste that cannot be reused. However, in recent years, fly ash has attracted the attention of researchers. It has been successfully synthesized in cement, concrete aggregates, roadbed construction, and other materials (Cetin et al. 2012), or used as an adhesive for soil improvement (Ramdas et al. 2021; Tian et al. 2022). Therefore, it is meaningful to investigate whether fly ash can solve the problem of EICP missing nucleation sites, that is, can fly ash be employed to treat desert sand to be improved in this study? To what extent can the addition of fly ash improve the mechanical properties of desert sand by EICP? At the same time, EICP, as a bio-curing technology, should be able to clarify whether it can be promoted in practical applications, and further exploration and testing are needed. The main components involved in EICP include urease, urea ($CO{\left( {N{H_2}} \right)_2}$) and calcium chloride ($CaC{l_2}$). Equations (1) and (2) list the chemical reaction equations involved.

1 $$CO{\left( {N{H_2}} \right)_2} + 2{H_2}O\buildrel {Urease} \over\longrightarrow 2N{H_4}^ + + C{O_3}^{2 - }$$

2 $$C{a^{2+}}+C{O_3}^{{2 - }}=CaC{O_3} \downarrow$$

In the present work, a series of laboratory experiments were conducted in order to assess the effect of tap water as a solvent on bio-cement variables, calcium carbonate morphology, precipitation efficiency, sediment and precipitate properties. The aim is to highlight the applicability of tap water in bio-cement, and the substitution of tap water for deionised water will extend the range of applications for bio-cement, especially for practical engineering applications where the transport of deionised water results in additional costs. Shear strength and permeability tests were used to methodically derive the pattern of change in the mechanical parameters of the amended soil. In addition, X-ray diffraction (XRD) and scanning electron microscope (SEM) test results were utilized to explain the mechanism of desert sand amelioration. The main work includes: firstly, the value of tap water in bio-cement was clarified, and the most suitable ratio of tap water as a solvent to prepare bio-cement under various influencing factors was investigated. Secondly, the characteristics of improved desert sand in terms of shear resistance and permeability were summarized. Finally, this work lays a certain research foundation for expanding the application scope and cost control of bio-cement in practical engineering, and also provides new ideas for interdisciplinary research between bioengineering, ecology and civil engineering.

## Materials and methods

### Desert sand (DS)

**Fig. 1:**
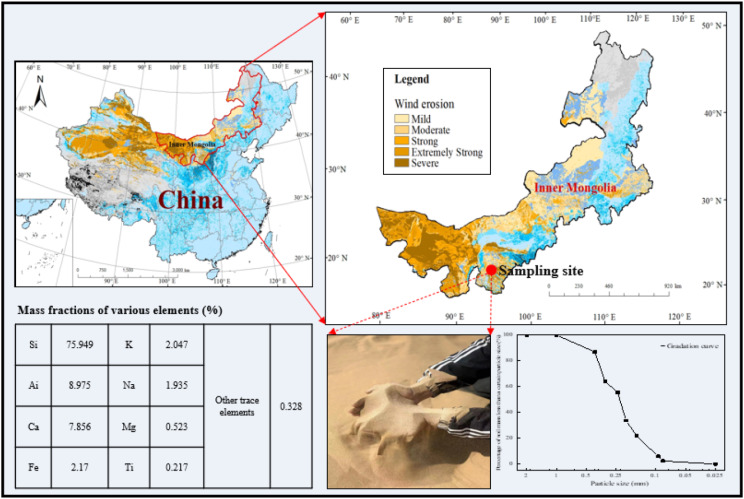
Sand sampling site in the Kubuqi Desert

Desert sand is formed under the action of wind erosion. Its characteristics mainly include small particle size (generally less than 0.25 mm), high alkali content, and large impurity content, which do not meet the standard of building sand. The desert sand used in this study was obtained from Kubuqi, China (see Fig. 1).

The sand in this area is very fine and free of impurities, and the main particle size is between 0.074 and 0.25 mm. Among various factors, *d*_*10*_, *d*_*30*_, *d*_*50*_, and *d*_*60*_ are obtained as 0.1, 0.15, 0.21, and 0.21, respectively, non-uniformity coefficient *C*_*u*_=2.1, curvature coefficient *C*_*c*_=1.07, and poor grading. In addition, the main physical and chemical properties have been presented in Table 1.

**Table 1:** Physical and chemical properties of the desert sand

Apparent density(g/cm^3^)	Stacking density (g/cm^3^)		Water content(%)		Clay content(%)	Fineness modulus	Chloride content(%)	Max dry density(g/cm^3^)	Optimal moisture content(%)
2.655	1.560	0.28		0.40		0.72	0.017	1.767	12

### Solutions

The solutions involved in this study include tap water, urease solution, urea solution, and calcium solution.

**(1) Tap water.** Basic indicators of tap water include pH 6.86, dissolved oxygen 10.99 mg/L, ammonia nitrogen 0.69 mg/L, and water hardness 142 mg/L.

**Fig. 2:**
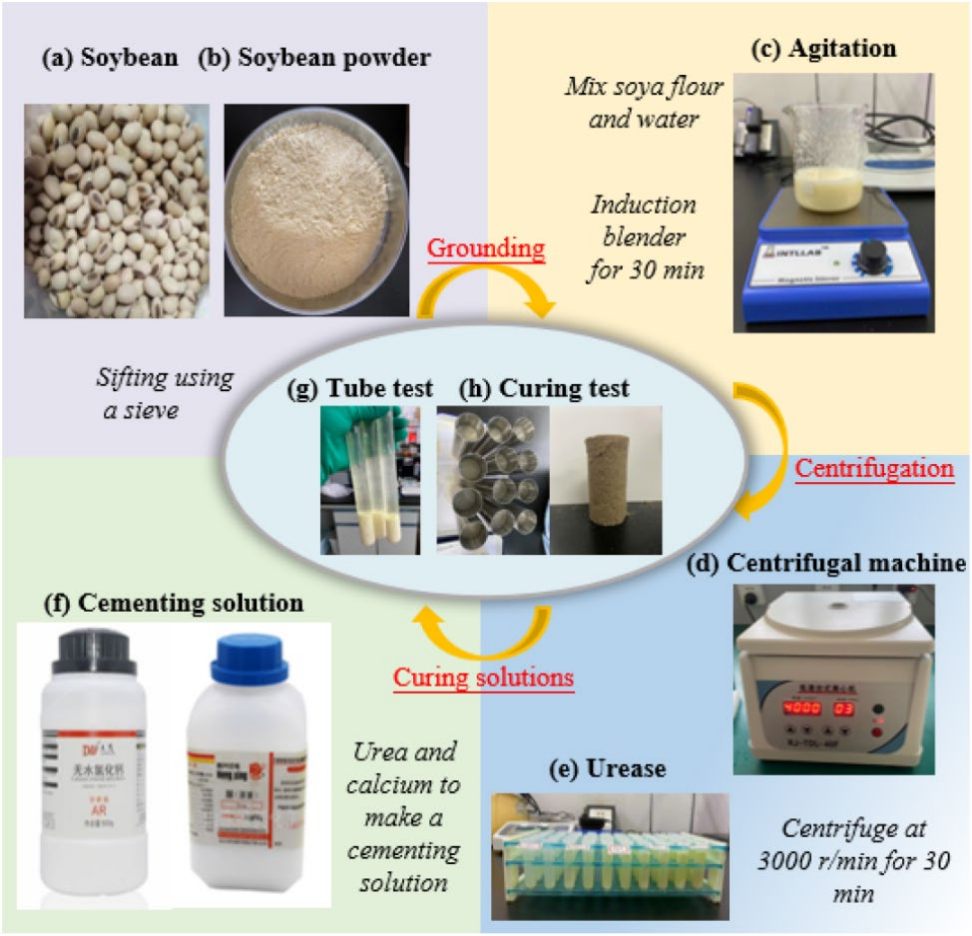
Preparation of the solution required for the test

**(2) Urease solution.** The urease used in this experiment was extracted from soybeans produced in Heilongjiang, China. After drying and grinding, soybeans were sieved by 0.150 mm to obtain soybean powder. Soybean powder and water were then appropriately mixed and stirred with an electromagnetic stirrer for 30 min. The mixture was transferred into a centrifuge tube by a pipette and centrifuged at 3000 r/min for 30 min. The filtrate can be filtered and collected, and the solution can also be allowed to stand for a while to collect the supernatant. The collected solution is soybean urease solution, and the extraction steps are illustrated in Fig. 2(a)-(e). The solution corresponds to the crude extract and should be kept in the refrigerator.

**(3) Cementing solution (CS).** The cementing solution in this work consists of a urea solution and a calcium solution, wherein the calcium solution is provided by calcium chloride.

### Fly ash (FA)

Fly ash is a product of coal combustion (Kotova et al. 2016). The appearance of fly ash is generally gray or blackish gray, and the higher the carbon content, the darker the color.

**Table 2:** Chemical compositions of fly ash

Chemical component	SiO_2_	Al_2_O_3_	CaO	Fe_2_O_3_	MgO	SO_3_	Other
Content(%)	50.40	27.45	6.73	5.90	2.72	2.9	3.9

According to various origins and sources, the physical properties of fly ash could be also different. Its density mostly varies in the interval of 2.1–2.6 g/cm^3^, particle size range is 0.5–300 μm, specific surface varies in the interval of 0.2–0.4 m^2^/g, and porosity is usually between 60 and 70% (Querol et al. 1997). Herein, the used fly ash is the secondary fly ash of Henan Huifeng New Materials Co., Ltd., where the water demand is 94%, the bulk density is 2.5 g/cm^3^, and the detailed chemical compositions are presented in Table 2.

### Tube tests

The test-tube experiments in this work aimed to find out the most suitable proportion of bio-cement under various influencing factors (ambient temperature, pH, urease mass concentration, urea concentration, calcium concentration, gum-enzyme ratio, curing time).

### Urease activity

#### (1) Urease concentration and temperature

In order to study the synergistic effect of urease concentration and temperature on urease activity (Whiffin et al. 2004), five different mass concentrations of urease solutions were prepared. Three ml of each concentration of urease solution was prepared and mixed with 27 ml of 1.0 mol/liter urea solution and then placed in a thermostatic incubator, set at different temperatures and kept at each temperature condition for 10 min before assessing the urease activity after monitoring the conductivity of each mixture using a conductivity meter.

### (2) Urea concentration and pH

Urea concentration and pH also had a synergistic effect on urease activity. Eight different concentrations of urea solutions were prepared and the pH of each concentration of urea solution was adjusted to different levels of 4.0, 5.0, 6.0, 7.0, 8.0, 9.0, 10.0, and 11.0. Then 27 ml of each combination of urea solution was mixed with 3 ml of 100 g/L urease solution, tested for electrical conductivity and calculated for urease activity under room temperature conditions (28 ± 2 °C).

### Calcium carbonate precipitation test tube

#### (1) Urease concentration

Urease solutions with mass concentrations of 20, 40, 60, 80, and 100 g/L were prepared. Each concentration of urease solution was mixed with 10 ml of cementing solutions with an equal volume, and the amount of calcium carbonate precipitation in each mixed solution was determined after 1*d* standing.

### (2) Urea concentration

Twenty urea solutions with molar concentrations ranging from 0.1 mol/L to 2.0 mol/L were prepared and mixed with 1 mol/L calcium chloride solution in equal volume, and finally with urease solution, and the amount of calcium carbonate precipitated from each mixed solution was determined after 1*d* of standing.

### (3) Calcium concentration

Eighteen types of calcium chloride solutions with different concentrations from 0.1 mol/L to 1.5 mol/L were prepared and eighteen types of calcium chloride solutions with different concentrations were mixed with 1 mol/L urea solution in equal volume to form eighteen types of urea solutions. In different cementing solutions, 10 ml of each cement solution was mixed with an equal volume of urease solution with a mass concentration of 100 g/L, and the amount of calcium carbonate precipitation in each mixed solution was determined after 1*d* standing.

### (4) pH of the consolidating fluid

Three kinds of urease solutions with different concentrations of 0.5, 1.0 and 1.5 mol/L were prepared, and mixed with calcium chloride solution in equal volume to form a cementing solution, and then each mixture was mixed with four kinds of cementing solutions with different pH values of 6.0, 7.0, 8.0 and 9.0, respectively, and the amount of calcium carbonate precipitated in each mixture was determined after 1*d* of resting.

### (5) Enzyme-glue ratio & curing time

The significance of enzyme-cement ratio is similar to the water-cement ratio in cementitious materials. In this study, we compared the calcium carbonate yield of different doses of urease solution reacted with the same concentration of cementing solution.

Meanwhile, a cementing solution with a concentration of 1 mol/L was prepared, and the same volume of cementing solution was mixed with urease solution, and then the amount of calcium carbonate precipitation after different curing days (1, 3, 5, 7, and 9 d) was measured.

Each of the above influences was tested at room temperature (28 ± 2 °C). Except for “(4) pH of the consolidating fluid”, the effect of pH value was not considered.

### Soil column tests

In this section, all samples were prepared using the mixing method. The solution ratios were selected by referring to “Sect. 2.4” to obtain the most suitable ratios.

### (1) DST

**Fig. 3:**
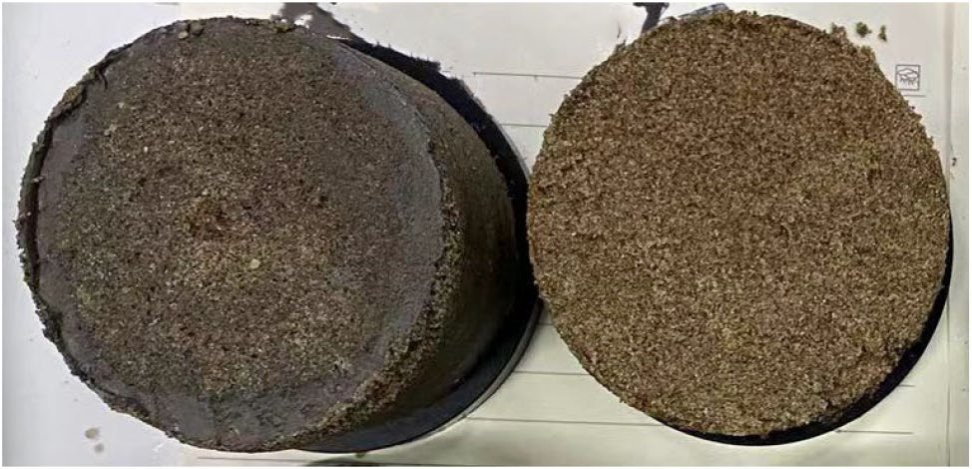
A specimen subjected to a direct shear test

The dimensions of the direct shear test specimens were 61.8 mm in diameter and 20 mm in height. To facilitate sample preparation and sample release, the sample preparation was carried out in a ring cutter with the bottom of the ring cutter sealed with plastic and the inner wall uniformly coated with petroleum jelly. The weight of the desert sand was 100 g and the pore volume was 38.78 cm^3^ (obtained by the volumetric method). Samples (labelled as F0) were prepared by mixing the consolidation solution and urease solution with desert sand at a solution volume 1.1 times the pore volume. Similarly, the same mass of desert sand was mixed with different amounts of FA (1%, 3%, 5%, 7%, 9%, 11% and 13%) to prepare samples (F1, F3, F5, F7, F9, F11 and F13). The desert sand, FA, cementing solution and urease solution were mixed homogeneously, compacted in layers, placed in moulds and removed after 7d of curing (see Fig. 3). The samples were subjected to DST tests at four stresses (i.e., 100, 200, 300, and 400 kPa) at a strain rate of 0.8 mm/min.

### (2) PT and CCS

The variable head penetration test is utilized to test the water conductivity of the cured sample. For the same specimen, the head change and time are recorded at predetermined intervals, then the water level of the header pipe is raised to the required height and the water temperature at the outlet is measured, and after several consecutive measurements the header pipe water level is raised to the initial height, and so on for many times, and the average value is calculated as the final value of the specimen. At the same time, parallel samples were set for all samples. The dimensions of the penetration test specimen are 61.8 mm in diameter and 40 mm in height (The sample is prepared in the same way as for DST samples).

The amount of calcium carbonate precipitation in the sample after curing is an important parameter to evaluate the curing level. The sample is weighed after the DST test and weighed again after the pickling test. The difference in weight before and after pickling is the amount of calcium carbonate precipitation that is obtained by processing.

### (3) XRD and SEM

To determine the distribution of calcium carbonate crystals and desert sand particles, a piece of solid sample was cut down and characterized by SEM. At the same time, an X-ray diffraction test was employed to assess the distribution of calcium carbonate after curing.

### Experimental results

#### Tube tests

##### (1) Urease activity

**Fig. 4:**
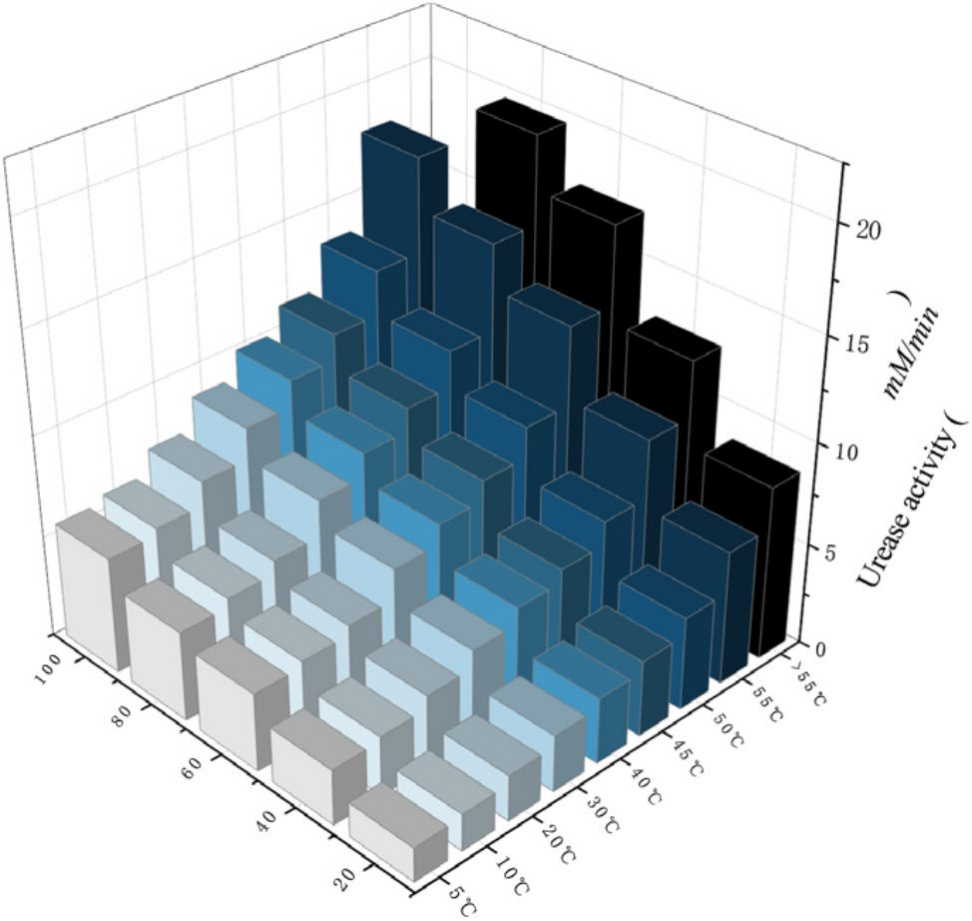
Urease activity subjected to various temperatures in the presence

Temperature affects the catalytic effect of the enzyme. Proper temperature control is able to maintain the high activity of the enzyme and thus raise the rate of the enzyme reaction. As illustrated in Fig. 4, as the temperature continues to increase, some “cuboids” will be missed, which indicates that the concentration of urease is deactivated at a certain temperature.

It can be seen that the urease solution is temperature tolerant. The urease activity before inactivation was almost linearly related to urease concentration and temperature.

**Fig. 5:**
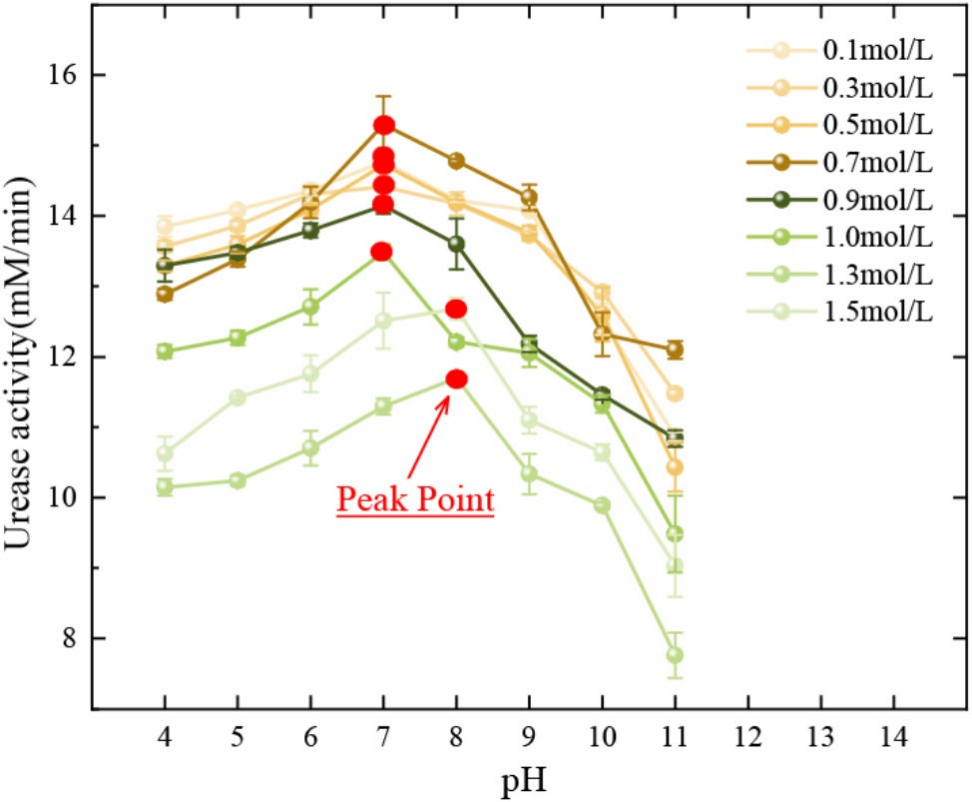
Urease activity under the influence of various pH levels in the presence

Figure 5 shows the trend of synergistic effect of pH and urea concentration on urease activity. The overall trend of urease activity was increasing and then decreasing and the optimum pH was obtained for different concentrations of urea solutions. However, for different concentrations of urea solutions, the optimum pH is 7 for concentrations in the range of 0.1 to 1.0 mol/L and 8 for concentrations greater than 1.0 mol/L.

Therefore, the appropriate urea concentration and urease concentration solution can be chosen according to the temperature and pH values of the actual engineering environment, which results in the best amount of urease activity, developing favorable conditions for improving the bio-cement curing level.

#### (2) Calcium carbonate precipitation

The theoretical value of calcium carbonate precipitation can be evaluated based on the following formula:

3 $$w=C \times V \times M$$

where *C* represents the concentration of cementing solution in moles per liter (mol/L), *V* is the volume of cementing solution in liters (L), and *M* denotes the molar mass of calcium carbonate (Neupane et al. 2013) which is equal to 100.09 g/mol.

### (1) Urease concentration

**Fig. 6:**
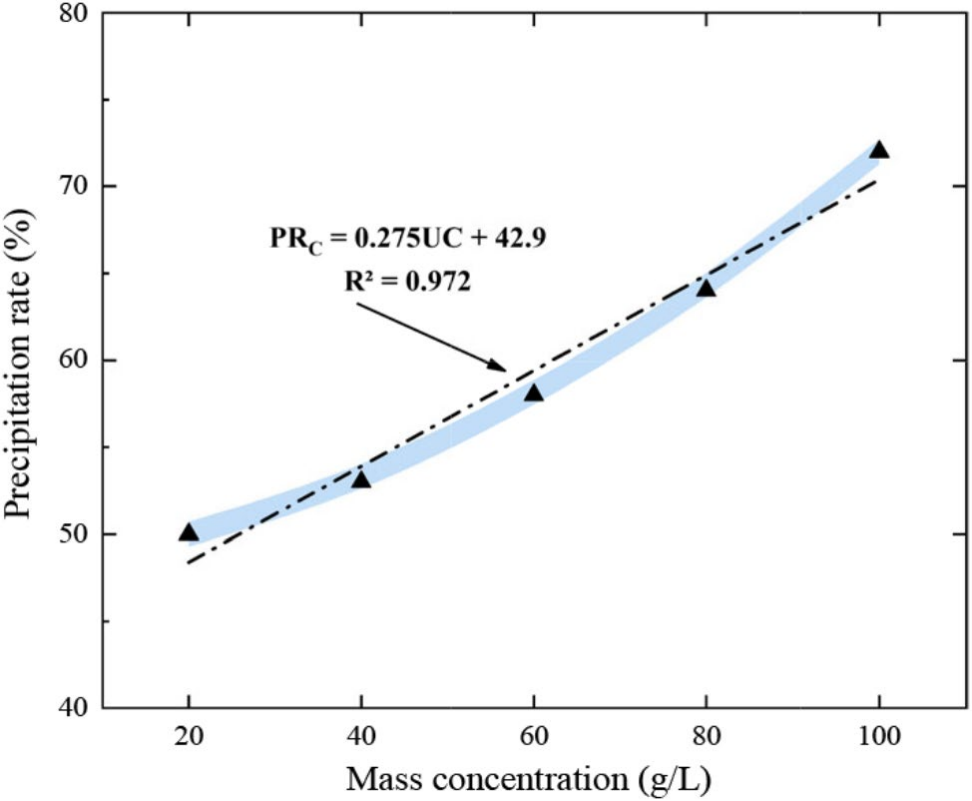
Relationship between precipitation rate of calcium carbonate and urease concentration

The variation curve of calcium carbonate precipitation rate with urease concentration has been presented in Fig. 6. The plotted results indicate that the amount of calcium carbonate precipitation has a positive correlation with the mass concentration of urease. However, choosing the right mass concentration of urease is useful for cost control (100 g/L is more appropriate). The results also reveal that the urease concentration and the calcium carbonate precipitation rate can be evaluated in the following form:

4 $$P{R_c} = {\raise0.7ex\hbox{${w'}$} \!\mathord{\left/{\vphantom {{w'} w}}\right.\kern-\nulldelimiterspace}\!\lower0.7ex\hbox{$w$}}$$

where $P{R_c}$ represents the precipitation rate of calcium carbonate, *w* is the theoretical precipitation amount of calcium carbonate, and *w* is the actual precipitation amount of calcium carbonate.

#### (2) Urea concentration

**Fig. 7:**
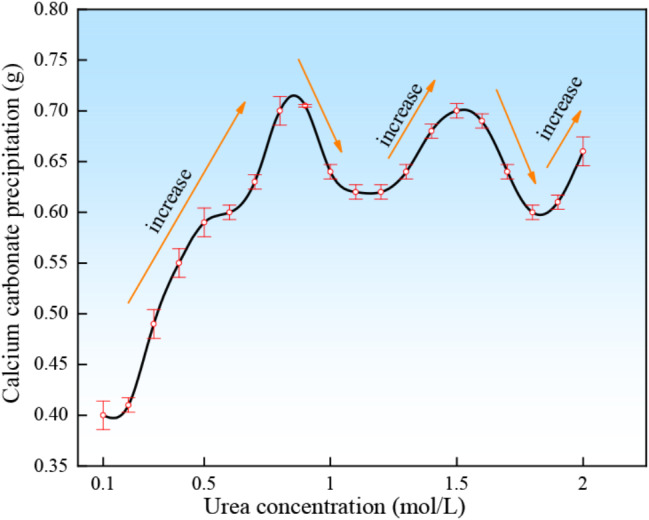
Calcium carbonate precipitation as a function of urea concentration

The change of calcium carbonate precipitation as a function of the urea concentration is presented in Fig. 7.

It can be seen from the figure that the amount of calcium carbonate precipitation increases linearly with the increase of urea concentration at 1.0 mol/L and the first precipitation peak is obtained at 1.0 mol/L concentration. As the concentration increased, the amount of calcium carbonate precipitation decreased in a small range, but the total amount of output remained more than 0.6 g. When the concentration of urea increased to 1.5 mol/L, the precipitation peak was obtained again and then decreased. It can be seen that the calcium carbonate precipitation is sufficiently desirable when the urea concentration is taken in the range of 1.0 mol/L to 1.5 mol/L.

#### (3) Calcium concentration

**Fig. 8:**
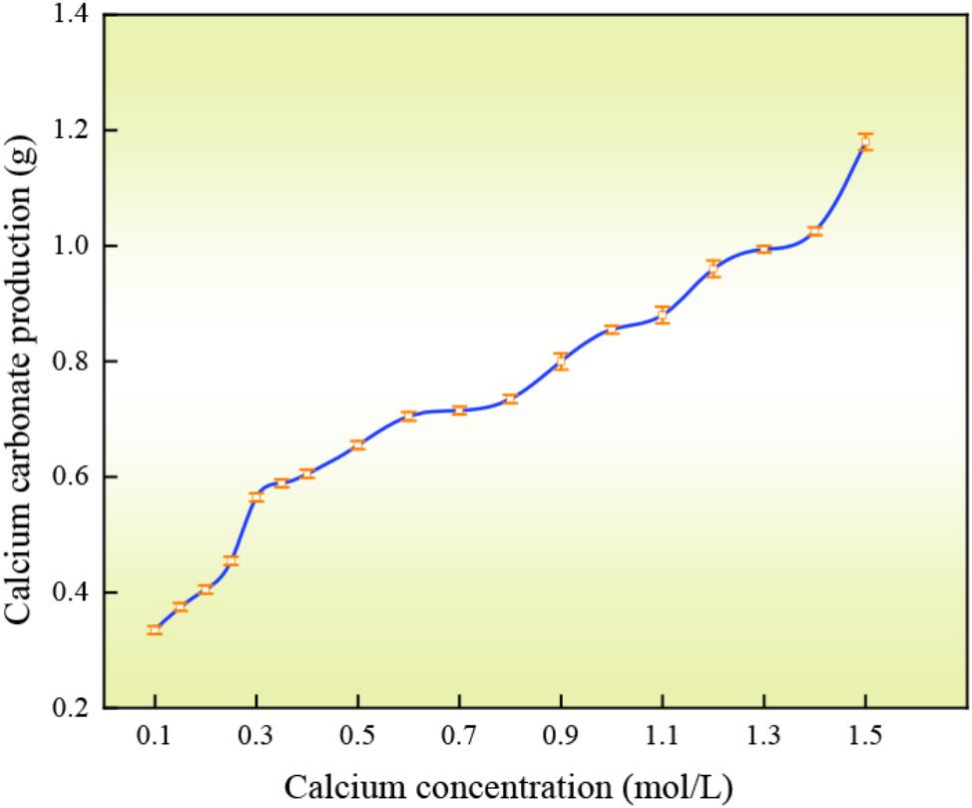
Variation of calcium carbonate precipitation with in calcium concentration

The trend of calcium carbonate precipitation under the influence of different calcium concentrations is shown in Fig. 8. There is no obvious peak in the amount of calcium carbonate precipitated due to different concentrations of calcium solution, and the amount of calcium carbonate precipitated is positively correlated with the concentration of calcium solution. Therefore, if we want to balance the cost and the ideal amount of calcium carbonate precipitation, it is more appropriate to control the concentration of calcium solution in the range of 1.0–1.5 mol/L, which is convenient for calculating and preparing the cementing fluid. Overall, the amount of calcium carbonate precipitation is essentially limited by the content (Trushina et al. 2016) (i.e., the content of urea in the cementing solution).

#### (4) pH of the consolidating fluid

**Fig. 9:**
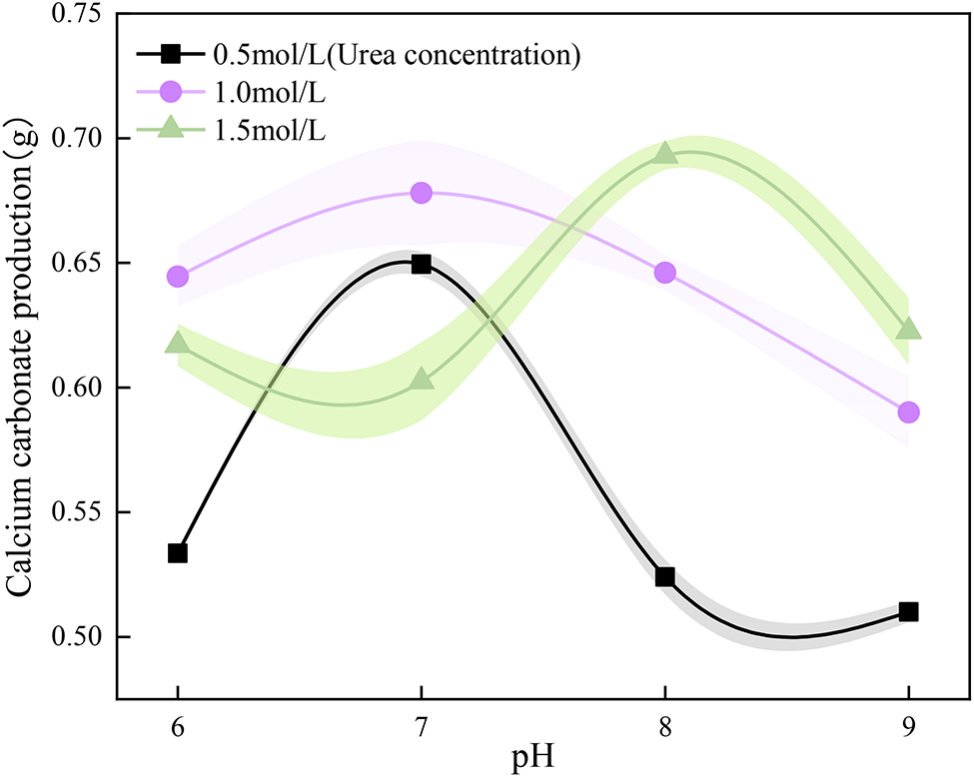
Effect of pH on the amount of calcium carbonate precipitated

The amount of calcium carbonate precipitation of the cementing solution prepared by high, medium, and low concentrations of urea solution under different pH conditions is shown in Fig. 9. The results indicate that there are peaks of calcium carbonate precipitation in three concentrations of cementing solution subjected to various pH conditions and the optimal pH value corresponding to each peak is different. The optimal pH value of the cementing solution with a concentration of 0.5 mol/L and 1.0 mol/L is 7.0, and the optimal pH value of the cementing solution with a concentration of 1.5 mol/L is 8.0. This finding also confirms the test results given in “Sect. 3.1”.

Therefore, it is necessary to consider the concentration of urea when setting the pH value of the cementing solution. When the concentration of urea is less than or equal to 1.0 mol/L, the pH value of 7.0 is more suitable. When the concentration of urea is greater than 1.0 mol/L, the pH value of 8.0 is more suitable. In practical engineering, the synergistic effect of the solution concentration with the ambient pH of the soil to be treated should be taken into account and the solution pH should be adjusted to obtain satisfactory settling values.

#### (5) Enzyme-glue ratio and reaction time

**Fig. 10:**
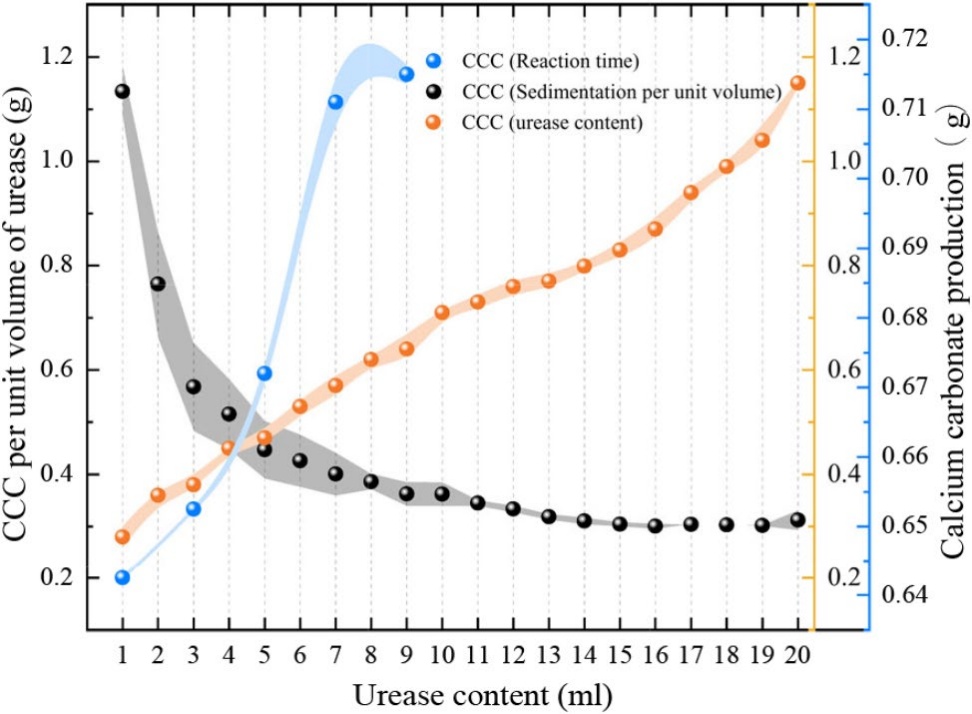
Effect of the enzyme ratio on the precipitation of calcium carbonate

By examining the amount of calcium carbonate precipitated per unit volume of urease solution (see Fig. 10), it can be seen that the amount of calcium carbonate produced per unit volume of urea solution decreases considerably at enzyme-to-glue ratios of 0.2 and 0.3.

By increasing the enzyme-gel ratio, the calcium carbonate yield per unit volume of urease solution decreases from a low enzyme-gel ratio to a high enzyme-gel ratio and gradually converges to a certain value. The enzyme-cement ratio in bio-cement is similar in significance to the water-cement ratio in concrete and is an important indicator of performance. It is clear from these results that blindly increasing the amount of urease does not increase the amount of calcium carbonate precipitated per unit volume of urease solution. The production of calcium carbonate under different reaction time conditions is shown in Fig. 10, where it can be seen that increasing the curing time promotes the amount of calcium carbonate precipitated, thus favouring the curing level. In the presence of calcium ions, more calcium carbonate precipitation is produced with time, i.e., the amount of calcium carbonate precipitated is positively correlated with the reaction time.

#### (6) Optimum ratio

The most suitable ratios of tap water as solvent under multifactorial conditions are presented in Table 3. It can be seen that the prepared urease solution showed good activity as well as the change rule under the influence of various factors. In conclusion, tap water can be used instead of deionised water in the bio-cement curing process, which extends the application of bio-cement curing technology to a certain extent.

**Table 3:** The optimal combination under multifactorial conditions

Urease activity test		Calcium carbonate precipitation test			
Temperature and tolerance	pH value (Urea concentration)	Urea concentrations	Calcium concentration	pH value of cementing fluid	Enzyme glue ratio
30℃(Strong)	pH = 7(0.5–1.0 mol/L) pH = 8(1.5 mol/L)	1.0 ~ 1.5(mol/L)	Linear growth	pH = 7(≤ 1.0 mol/L) pH = 8(>1.0 mol/L)	0.2–0.3

## (3) Solidification level with fly ash addition

In this section, the most suitable ratio for preparing the sample is obtained through the above test, that is, 1 mol/L urea solution and 1 mol/L calcium chloride solution are made as a cementing solution and the pH value of the solution is set equal to 7. A mass concentration of urease solution of 100 g/L is considered; the ambient temperature is controlled at 30 °C (± 2 °C), which is the sample curing temperature. It is worth mentioning that all the stages of soil sample preparation in this article have been completely performed by the replacement of deionized water with tap water.

### (4) Shear stress-displacement relationship

**Fig. 11:**
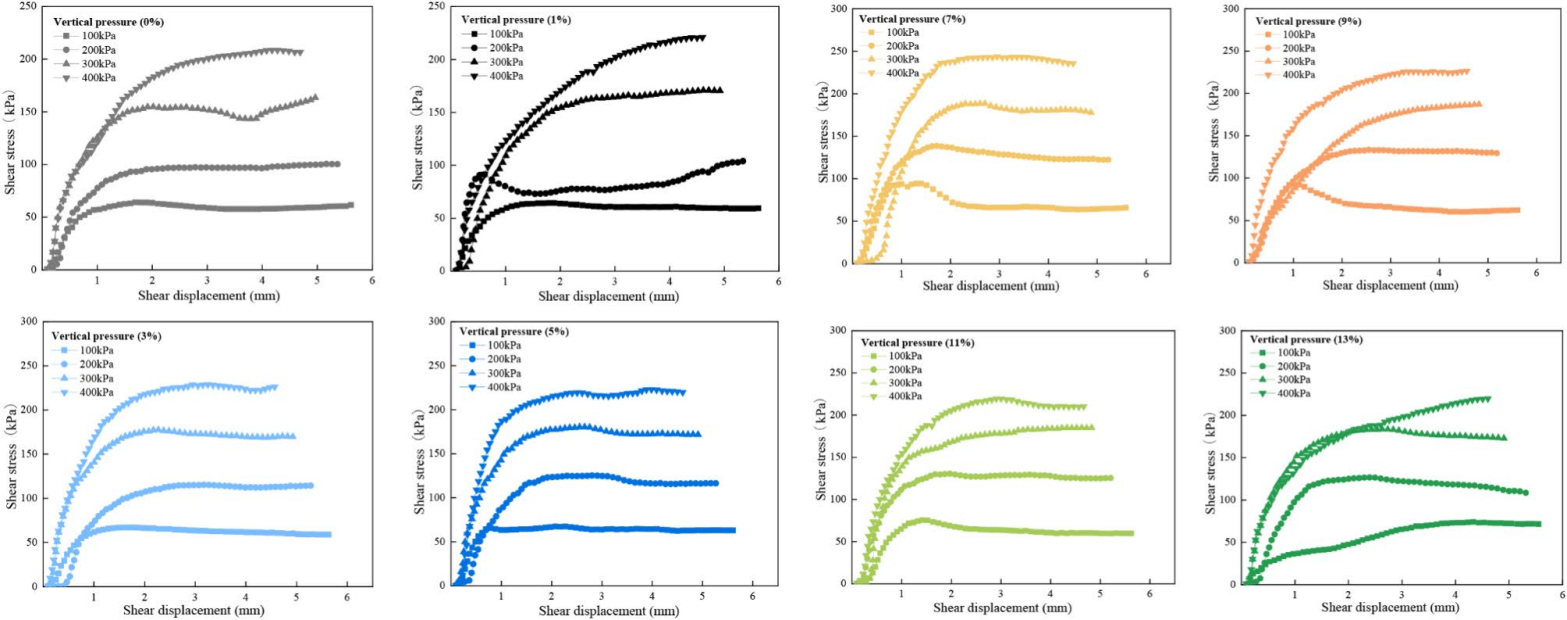
Shear stress-shear displacement curves in the presence of various FA dosages and vertical pressures

The direct shear test results of desert sand samples treated with EICP-FA are illustrated in Fig. 11. This figure shows the change response of desert sand samples after mixing subjected to four normal stresses (i.e., 100, 200, 300, and 400 kPa) during the shearing process. In all normal stress conditions, the shear stress level of the sample after mixing is higher than the undoped sample, and with the increase in content, the shear strength level also increases in the same proportion. This result can be attributed to the friction of the sample under the action of a higher-pressure limit, and the contribution of the shear strength is dominant.

Meanwhile, desert sand, fly ash, and calcium carbonate contribute to cementing each other to form aggregates, preventing the movement of desert sand particles, which promotes the bite force and friction force. The highest peak shear strength (F7) was 37.9% higher than that of the unadded admixture sample (F0).

The smoothness of the shear stress-strain curve in the figure also indicates the roughness of the shear surface of the sample. It should be noted that when the amount of fly ash is more than 9% (226.37 kPa), the shear strength of the sample is significantly lower than the sample mixed with 7% (243.51 kPa) fly ash. It should be noted that the shear strength values of the desert sand without added fly ash and at the same time without bio-cement under four normal pressures are 45.12 kPa, 79.81 kPa, 101.45 kPa and 125.65 kPa, with cohesion of 0 and internal friction angle of 18.26°, respectively. All the data sets, including the friction angle, are summarized in Table 4.

**Table 4:** Change in cohesion and angle of internal friction with dosage

Dosages/%	σ/ kPa	τ_peak_/ kPa	c_peak_	ϕ_peak_/ °	Dosages/%	σ/ kPa	τ_peak_/ kPa	c_peak_	ϕ_peak_/ °
0	100	64.08	10.21	20.97	7	100	94.2	41.78	35.60
	200	100.45				200	138.58		
	300	163.41				300	188.4		
	400	208.58				400	243.51		
1	100	64.4	13.96	24.19	9	100	92.92	39.07	33.51
	200	103.97				200	133.45		
	300	170.94				300	186.96		
	400	220.76				400	226.37		
3	100	66.8	20.13	29.08	11	100	75.46	38.34	33.91
	200	115.19				200	130.57		
	300	177.5				300	185.03		
	400	228.77				400	219.48		
5	100	67.45	29.63	32.50	13	100	74.01	38.66	33.41
	200	125.28				200	126.56		
	300	180.39				300	183.59		
	400	223				400	219.8		

This phenomenon of low shear strength in the presence of high fly ash content is due to excessive distribution of fine particles in the sample, which makes calcium carbonate fairly unable to form effective cementation between particles (Tian et al. 2022). Therefore, excessive fly ash content causes the sample to lose some of its strength. If obtaining a high-strength sample is of main purpose, the content should be appropriately controlled. In this research work, the amount of fly ash of 7% is able to achieve the goal of improving the shear strength of the sample.

### (5) Permeability

**Fig. 12:**
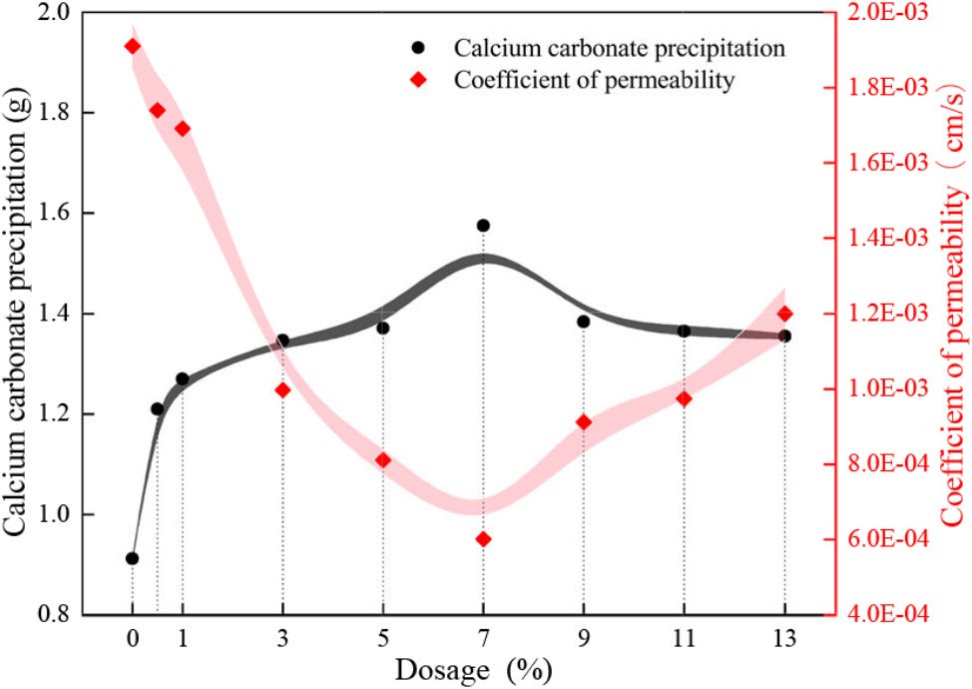
Permeability coefficient and calcium carbonate precipitation after solidification

Figure 12 shows the permeability and precipitation rate of calcium carbonate in the presence of different dosage conditions. The permeability coefficients of the EICP-FA treated desert sand samples ranged from 6.01 × 10^− 4^ to 17.4 × 10^− 4^ cm/s. The permeability coefficients decreased by 8.9–68.5% compared to the samples without added fly ash (19.1 × 10^− 4^ cm/s), with a decreasing trend with increasing calcium carbonate precipitation. It is worth noting that the permeability coefficient of desert sand without the addition of fly ash and also without the addition of bio-cement is 22.5 × 10^− 4^ cm/s.

The changing trend of calcium carbonate precipitation was opposite to the trend of permeability coefficient. The calcium carbonate content of the EICP-FA treated samples increased by 72.7% compared to the samples treated without additives.

#### (6) Microstructures

**Fig. 13:**
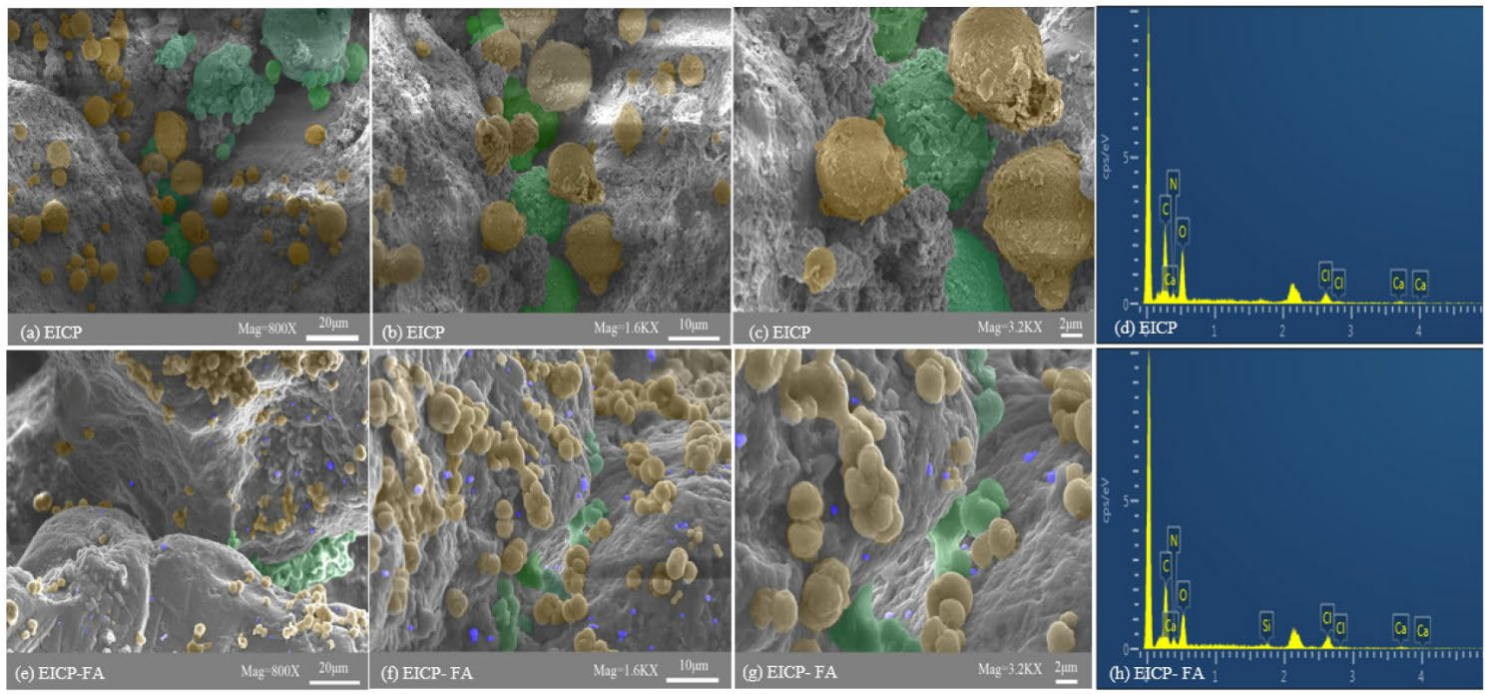
SEM-EDS images of soil particles after EICP and EICP combined with fly ash treatment

The samples used for microdetection were taken from the post-damage specimens of the straight shear test, where an admixture doping of 7% was used for microstructural testing of the EICP-FA modified desert sand. Through scanning electron microscope images of the cured samples, it can be seen that calcium carbonate precipitate is formed (see Fig. 13), revealing the difference between EICP and EICP-FA treated samples. The calcium carbonate of the EICP-treated samples is only dispersed on the surface of the soil particles or a small amount is distributed between the soil particles (see Fig. 13(a)-(c)).

After EICP-FA treatment, the agglomeration of calcium carbonate crystals appeared in the samples and the aggregates were densely distributed (see Fig. 13(e)-(g)). The distribution of calcium carbonate obtained by EICP-FA was wider than that of EICP, and the particle size of calcium carbonate crystals was relatively small. This is because the more viscous the solution, the smaller the particle size of the calcium carbonate crystals (Nemati et al. 2005). The yellow and green rounded particles shown in Fig. 13 are all precipitates from biomineralisation, i.e. calcium carbonate crystals, where the yellow calcium carbonate crystals encapsulate the soil particles and form an accumulator, and the green calcium carbonate crystals act as a bridge at the soil particles. FA is marked in purple.

Figure 13(d) and (h) show the elemental spectra of the bio-cement treated samples after energy spectroscopy, with each peak in the spectrum indicating the relative concentration of the element. The main chemical compositions of the FA include O, Si, and small amounts of Ca (Table 2). Samples treated with EICP-FA contained silica compared to specimens modified only with bio-cement.

Therefore, the contribution of FA and cementing fluid flow is capable of increasing the contact area between desert sand particles, providing nucleation sites for calcium carbonate, and then compensating for the shortcomings of EICP technology. On the other hand, this also explains why the DST and permeability resistance of the sample after EICP-FA treatment is superior to EICP. When the sample becomes dehydrated and hardened, the particles wrapped in the cementing fluid interact more closely, which is reflected in the improvement of the UCS and permeability resistance.

**Fig. 14:**
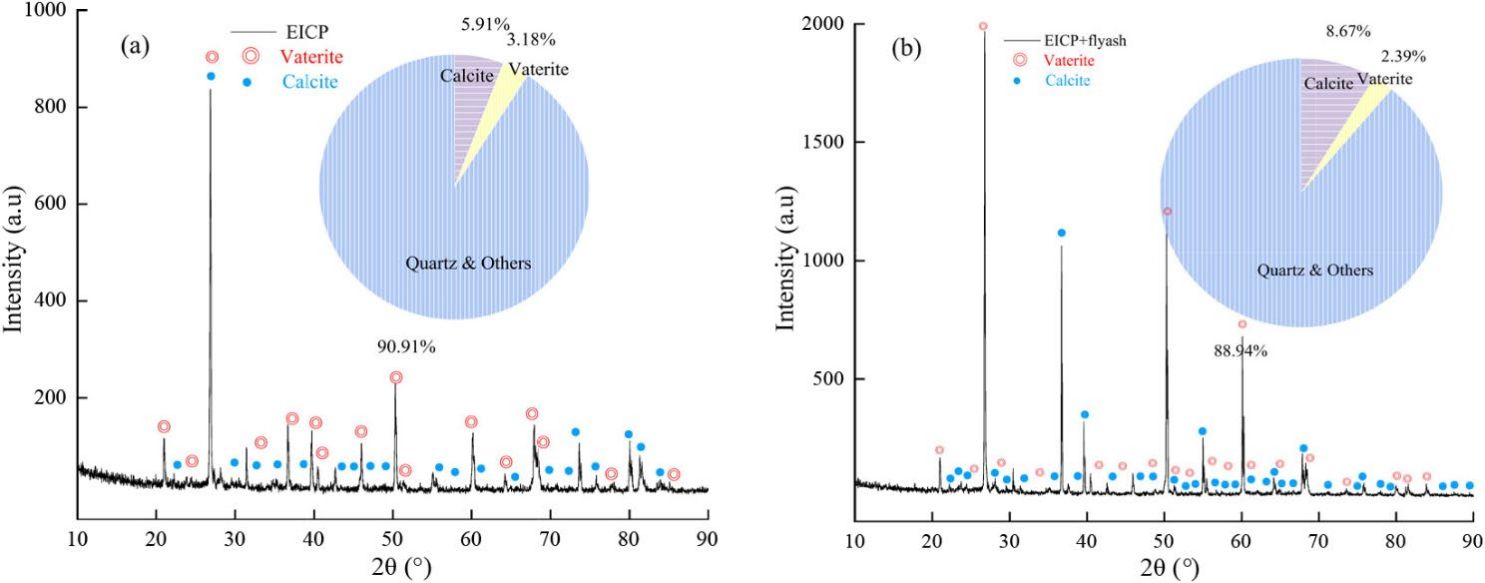
SEM images of soil particles after EICP and EICP-FA treatment

XRD is an efficient tool commonly used to identify and distinguish a variety of minerals (Nafisi et al. 2019), and the peaks and intensities of various types of calcium carbonate crystals, such as vaterite, calcite, and aragonite (Oral et al. 2018). The achieved results revealed that calcium carbonate crystals appeared in the samples treated with EICP and EICP-FA (see Fig. 14). The maximum strength of calcium carbonate crystals formed in the samples treated with EICP-FA was higher than that of the samples treated with EICP alone, indicating a higher content of calcium carbonate. Further comparative analysis showed that, compared to the samples treated with EICP alone, the diffraction peaks in the samples treated with EICP-FA were substantially increased, indicating that EICP-FA produced a large amount of calcite. Such a fact essentially explains why the strength of the samples after adding FA is higher than that of the FA-free samples.

As shown in Fig. 14(a), the main mineral compositions of EICP are calcium carbonate and quartz, and the generated calcium carbonate mainly consists of vaterite and calcite, accounting for 3.18% and 5.91%, respectively. As shown in Fig. 14(b), the main mineral composition and generated calcium carbonate of EICP-FA are consistent with that of EICP, in which the generated vaterite and calcite account for 2.39% and 8.67%, respectively.

## Discussion

**Fig. 15:**
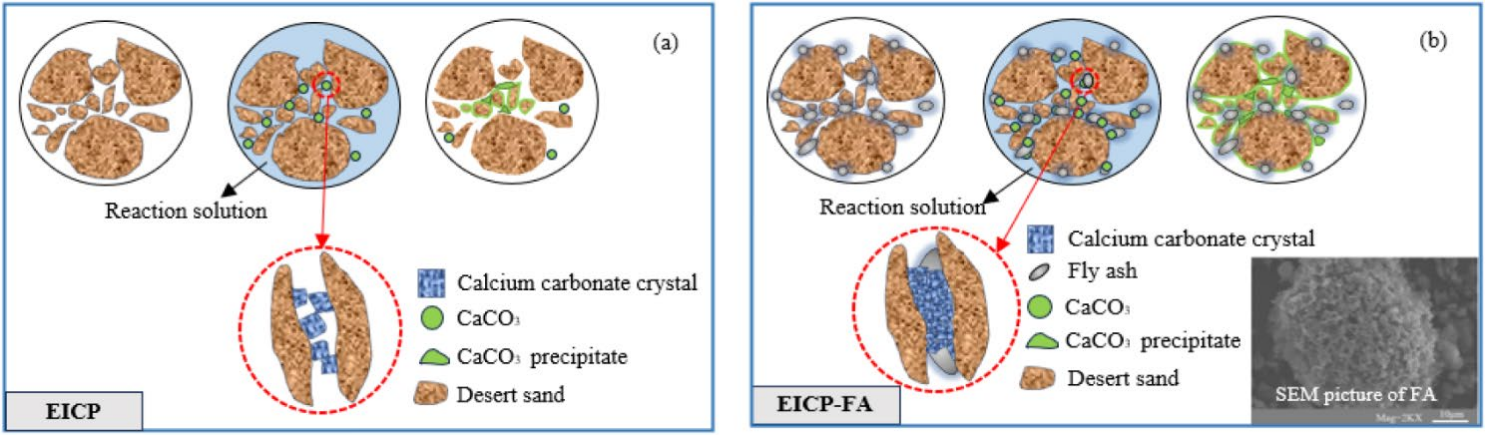
Curing mechanism diagrams for: (**a**) EICP, (**b**) EICP-FA

Herein, the possibility of improving desert sand through simple mixing and compaction, without the need for a bioreactor, was thoroughly examined and proved. In addition, the value of the cost minimization parameter was determined. The use of soya flour as a source of urease has been reported to be more economical than commercial urease, which is expensive (20 euros per gram, based on indicative prices from commercial suppliers for 2021) and uneconomical especially for large-scale practical engineering applications (Isaac et al. 2021). If commercial urease is used, it will be the most expensive component of the chemicals used in bio-cement. Furthermore, the use of plant urease is able to avoid the limitations of oxygen input involved in MICP bacterial urease and makes EICP-FA more practical. According to the test results, the success of EICP-FA in improving desert sands is mainly attributed to the following aspects:

(1) In general, there is no direct relationship between soil permeability and strength, but both are affected by pore filling. The denser the filling, the worse the water and air permeability of the soil, and the stronger the anti-permeability. When the particle arrangement is reorganized when disturbed by external forces, the spatial change is small and the contact surface between particles is large, resulting in high shear strength. Pore filling in bio-cement amended soils is achieved by the continuous accumulation and bridging of calcium carbonate. The results of anti-permeability and SEM are proof of this process, soil porosity has a great influence on its resistance in soil. Therefore, reducing pores in samples treated with EICP-FA is one of the reasons for increasing its strength. Losini (Losini et al. 2021) investigated the effects of natural additives and biopolymers on soil structure and found that both natural additives and biopolymers are capable of improving the internal pore structure of the soil, which influences the thermal conductivity, infiltration properties, and mechanical properties.

(2) The presence of FA powder provides nucleation sites that promote the growth of calcium carbonate crystals and provide better binding between soil particles. On the one hand, the generated calcium carbonate crystals are spheres with smooth surfaces, while the fly ash particles have loose and porous surfaces with strong adsorption (Fig. 15(b)), which facilitates nucleation due to greater friction with the calcium carbonate crystals. On the other hand, Ca^2+^ in the solution is preferentially adsorbed to the fly ash surface, and carbon dioxide hydrolysis generates CO_3_^2−^ which immediately combines with Ca^2+^ on the fly ash surface, crystallising on the fly ash surface to generate a homogeneous cladding layer. Because there are silicon-oxygen bonds on the surface of FA, they become sites for the adsorption of calcium carbonate crystals. At the same time, the silicon-oxygen bonds have a strong polar effect. They produce polarization when exposed to water and are negatively charged, adsorbing calcium ions and other cations (Bayat et al. 2002). When the Ca^2+^ adsorbed on the surface of fly ash reaches saturation, there are a lot of free Ca^2+^ in the solution combined with CO_3_^2−^ to generate free calcium carbonate crystals and continue to increase in the formation of stacking, i.e., the occurrence of non-uniform nucleation, thus forming a “bridge” between the desert sand and fly ash. If the concentration of calcium solution in the cement is high enough, after mixing with fly ash, the concentration of reactants is also high, which leads to a certain degree of inhibition of hydrolysis, calcium carbonate crystals generated by the accumulation of obstacles, calcium carbonate crystals between the phenomenon of “wrapping”, the formation of “agglomerates “, this makes the EICP-FA solution is more viscous after mixing with fly ash.

(3) The integrity and stability of the desert sand was ultimately enhanced by the addition of FA to the EICP cementing fluid. Evaporating during the moisture curing process, the dehydration process increases the bond strength and brings the particles closer together, thus compressing the pore space (see Fig. 15). Having shorter bonding chains makes them more resistant to external forces, so geotechnical properties are improved during the drying process. The resulting consolidation fluid binds the desert sand together, forming a strong structure and increasing cohesion, thus improving the mechanical properties of the desert sand. Apart from fly ash, there are other admixtures used for nucleation, including skimmed milk powder (Martin et al. 2021), brown sugar, glutinous rice flour, xanthan gum (Wu et al. 2020; Tariq et al. 2022), lignin (Zhang et al. 2022), sodium-based montmorillonite (Yuan et al. 2022), guar gum (Hamdan et al. 2016), biopolymers (Refaei et al. 2020), polychitosan (Nawarathna et al. 2019), and others.

## Conclusions

In this work, a novel approach of bio-cement (Enzyme Induced Carbonate Precipitation, EICP) combined with fly ash (FA) is used to improve the shear strength and permeability of desert sand. Here, FA is regarded as an environmentally friendly waste recycling material and is synergistically improved with bio-cement, which provides a good idea for the cross research and application of bioengineering with ecology and civil engineering. We used tap water as a solvent to prepare all the solutions and analyzed the most suitable ratio of bio-cement under the influence of multiple factors. The successful application of tap water in bio-cement and the quantitative results of the in-house direct shear test and permeability test point the way for the application of desert sand in infrastructure engineering. The main results are summarized as follows:

(1) A new improvement method, EICP-FA, was applied and proved to enhance the shear strength and permeability of desert sand better than that treated with EICP only. EICP-FA is environmentally friendly and highly efficient, and it has a special application prospect in the utilization of desert sand and infrastructure construction in desert areas.

(2) The urease solution prepared with tap water is highly temperature resistant. The optimum pH was 7.0 at urease solution concentrations of 0.1 to 1.0 mol/L. At concentrations greater than 1.0 mol/L, the optimum pH was 8.0, which was related to the precipitation process of calcium carbonate in cementing solutions of different pH values. The precipitation amount of calcium carbonate was positively correlated with the mass concentration of urease solution, urea concentration, calcium concentration and reaction time. The concentration of urease can be controlled in the range of 1.0–1.5 mol/L to achieve the desired amount of precipitation. A calcium concentration range of 1.0–1.5 mol/L is desirable. The enzyme-gel ratio continued to increase as the amount of calcium carbonate produced per unit volume of urease solution gradually approached a value from low to high. Tap water treatment is more effective in reducing costs (Martin et al. 2020).

(3) The effect of increasing common parameters such as shear strength and water permeability was studied in depth. Both shear strength and permeability coefficient showed an increasing and then decreasing trend with the increase of fly ash dosage. The optimum value occurs at a fly ash content of about 7%. Specifically, the highest peak shear strength increased by 37.9%, the permeability coefficient decreased by 8.9 -68.5%, and the calcium carbonate content increased by 72.7% as compared to the samples without additives.

(4) The microstructure analysis revealed that the distribution of calcium carbonate in EICP-treated desert sand was irregular. However, the addition of FA provided nucleation sites for calcium carbonate crystal aggregation, which was beneficial for improving the mechanical properties of desert sand. The XRD-based analysis indicated that the crystalline form of calcium carbonate was calcite and aragonite in the EICP-FA treated desert sand, which was the most stable crystalline form.

### Electronic supplementary material

Below is the link to the electronic supplementary material.

Supplementary Material 1

## Data Availability

The data will be available on the reasonable request.
