# Supplementary figures and images for: An experimental study on the curing of desert sand using bio-cement
Source: Bioresour Bioprocess. 2024 Jul 20;11(1):72. doi: 10.1186/s40643-024-00788-y (PMC11264504; doi:10.1186/s40643-024-00788-y)

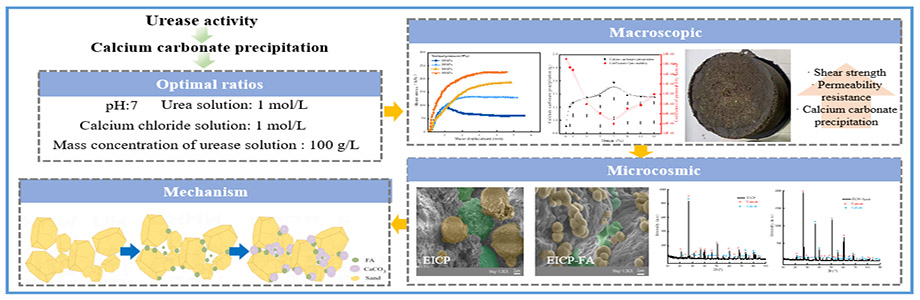

Supplement: Supplementary file 1 — Supplementary Material 1 [file 40643_2024_788_MOESM1_ESM.jpg]
